# Performance Evaluation of EEG Based Mental Stress Assessment Approaches for Wearable Devices

**DOI:** 10.3389/fnbot.2021.819448

**Published:** 2022-02-04

**Authors:** Ubaid M. Al-Saggaf, Syed Faraz Naqvi, Muhammad Moinuddin, Sulhi Ali Alfakeh, Syed Saad Azhar Ali

**Affiliations:** ^1^Electrical and Computer Engineering Department, King Abdulaziz University, Jeddah, Saudi Arabia; ^2^Center of Excellence in Intelligent Engineering Systems (CEIES), King Abdulaziz University, Jeddah, Saudi Arabia; ^3^Department of Electrical & Electronics Engineering, Center for Intelligent Signal and Imaging Research, Universiti Teknologi PETRONAS, Bandar Seri Iskandar, Malaysia; ^4^Department of Internal Medicine, Child and Adolescent Psychiatrist, Faculty of Medicine, King Abdulaziz University, Jeddah, Saudi Arabia

**Keywords:** stress-assessment, computer-aided diagnosis (CAD), machine learning, convolutional neural network, feature extraction, real time, sliding window, rehabilitation

## Abstract

Mental stress has been identified as the root cause of various physical and psychological disorders. Therefore, it is crucial to conduct timely diagnosis and assessment considering the severe effects of mental stress. In contrast to other health-related wearable devices, wearable or portable devices for stress assessment have not been developed yet. A major requirement for the development of such a device is a time-efficient algorithm. This study investigates the performance of computer-aided approaches for mental stress assessment. Machine learning (ML) approaches are compared in terms of the time required for feature extraction and classification. After conducting tests on data for real-time experiments, it was observed that conventional ML approaches are time-consuming due to the computations required for feature extraction, whereas a deep learning (DL) approach results in a time-efficient classification due to automated unsupervised feature extraction. This study emphasizes that DL approaches can be used in wearable devices for real-time mental stress assessment.

## 1. Introduction

Mental stress is the most common cause of psychological and physiological disorders in the human body. Stress is the response of a body when the brain experiences external burden. The human body functions abnormally under stress because it perceives that its resources have been compromised due to alien conditions. This initiates a chain reaction that might affect the normal functioning of essential organs (Rozanski et al., 1999). Mental stress can arise due to various situations or conditions. The factors that might induce stress varies for different people. School, workplace, social meeting, and presentation are common places or situations in which people tend to get easily stressed. Hence, stressful conditions are independent of gender, age, or occupation (McEwen, 2007). Apart from deteriorating cognition, stress might lead to chronic and irreversible brain damage. Immunological disorders are often linked to mental stress when a person is unable to handle it Yaribeygi et al. (2017).

Therefore, early detection of mental stress is crucial to avoid the detrimental effects of progression. Detection of mental stress involves different levels of challenges. The conventional face-to-face session-based method requires an expert to evaluate the level of stress based on a set of questionnaires (Dise-Lewis, 1988; Koh et al., 2001; Gomathi et al., 2014). However, such an assessment is possible only when someone acknowledges the consequences of mental stress and consults an expert for its treatment (Yorozu et al., 1987; Gomathi et al., 2014). Additionally, the social stigma associated with visiting a psychiatrist for mental evaluation induces situations such as dodging questions or providing dishonest answers, resulting in inaccurate assessment and inappropriate treatment. Therefore, the prolonged inability to handle stress leads to severe physical or physiological disorders.

To resolve the issue of misdiagnosis due to erroneous answers and to address the social stigma associated with visiting a psychiatrist, an intelligent mental assessment method should be developed to assist the practitioners and provide a self-assessment tool. The method should be accurate and reliable, and it should have practical applications (Naqvi et al., 2020). Multiple studies have attempted to overcome the challenges of the conventional face-to-face stress assessment method using various modalities to detect intrinsic changes in a body during a stressful session (Naqvi et al., 2020) use a deep learning (DL) approach to identify mental stress. Sharma et al. (2018) applied machine learning (ML) approaches to distinguish between stress and non-stressful electroencephalography (EEG) patterns, the study uses a support vector machine (SVM) with the highest accuracy. Subhani et al. (2016) also use statistical analysis to highlight the various locations that are affected during the mental stress period. Xia et al. (2018) classify the mental stress patterns into different levels. Al-shargie et al. (2015) also employ a ML approach to identify mental stress between four levels. Hu et al. (2015) and Al-Shargie et al. (2017) use different sensors to identify physiological changes and assessment of mental stress through which the patterns are classified. Arrighi et al. (2000) and Zhang et al. (2019). In these studies, mental stress assessment is performed through the analysis of blood flow and using fMRI. However, these methods are unviable because they are invasive or costly. These methods include, EEG (Saidatul et al., 2011; Al-shargie et al., 2015; Hu et al., 2015), functional near-infrared spectroscopy (fNIRS) combined with EEG (Al-Shargie et al., 2017), functional MRI (fMRI) (Zhang et al., 2019), and positron emission tomography (PET) (Arrighi et al., 2000). The most appropriate modality to fulfill the parameters of a wearable real-time device is EEG. It provides high-temporal information, which is essential to assess mental stress and further spatial information can be easily extracted using electrodes attached to the head. Additionally, the cost of an EEG-based system is comparatively low and it is easy to implement. Therefore, it is suitable for wearable devices. There are two types of ML techniques used for EEG-based mental stress assessment. The first type employs manual feature extraction and classification using conventional ML techniques such as SVM (Al-shargie et al., 2015), decision tree (DT) (Naqvi et al., 2020), and logistical regression (LR), the methods that use ML approaches usually lacks the ability to perform in real-time and due to the manual feature identification and extraction, the learning capability of the model suffers hence performing with low accuracy. The second type employs automatic feature extraction and classification through DL techniques such as convolutional neural network (CNN). It is crucial to perform an analysis of the computation time required for stress assessment to select an appropriate method for a wearable device for real-time assessment. However, a study that analyzes the computation time of various EEG-based assessment methods for real-time implementation has not been conducted. Therefore, in this study, the time consumption of different EEG-based mental assessment methods are compared for real-time implementation scenarios.

## 2. Methodology

The accuracy and the time required for assessment are essential parameters that are used to evaluate the performance of a method for its compatibility with real-time systems. To select suitable algorithms for a wearable mental stress assessment device, several ML and DL techniques were compared in terms of accuracy and computation time. ML approaches, such as SVM, DT, and LR, were considered and compared with that of CNN as a DL technique. These techniques have demonstrated high accuracy of stress assessment in previous studies.

ML and DL are slightly different in terms of process flow. However, they operate on the principle of feature extraction and then the classification of these features.

### 2.1. Process for ML and DL

In this research, the time required for stress assessment is a crucial factor. Therefore, the processes of manual signal cleaning are avoided because such processes do not allow the operation of a system in real-time. Raw data is passed through a signal filtration process which extracts a band and filters other irrelevant bands and noises. After the process of filtration, significant features are extracted that are used for the training and classification of stress patterns. The hardware used to compute and compare the response times for all the techniques applied, DELL-OPTIPLEX 990 with hardware configuration of 24 GB RAM and Intel i7 3.4 GHz 2nd Gen. CPU was used throughout this study.

**Figure 1:**
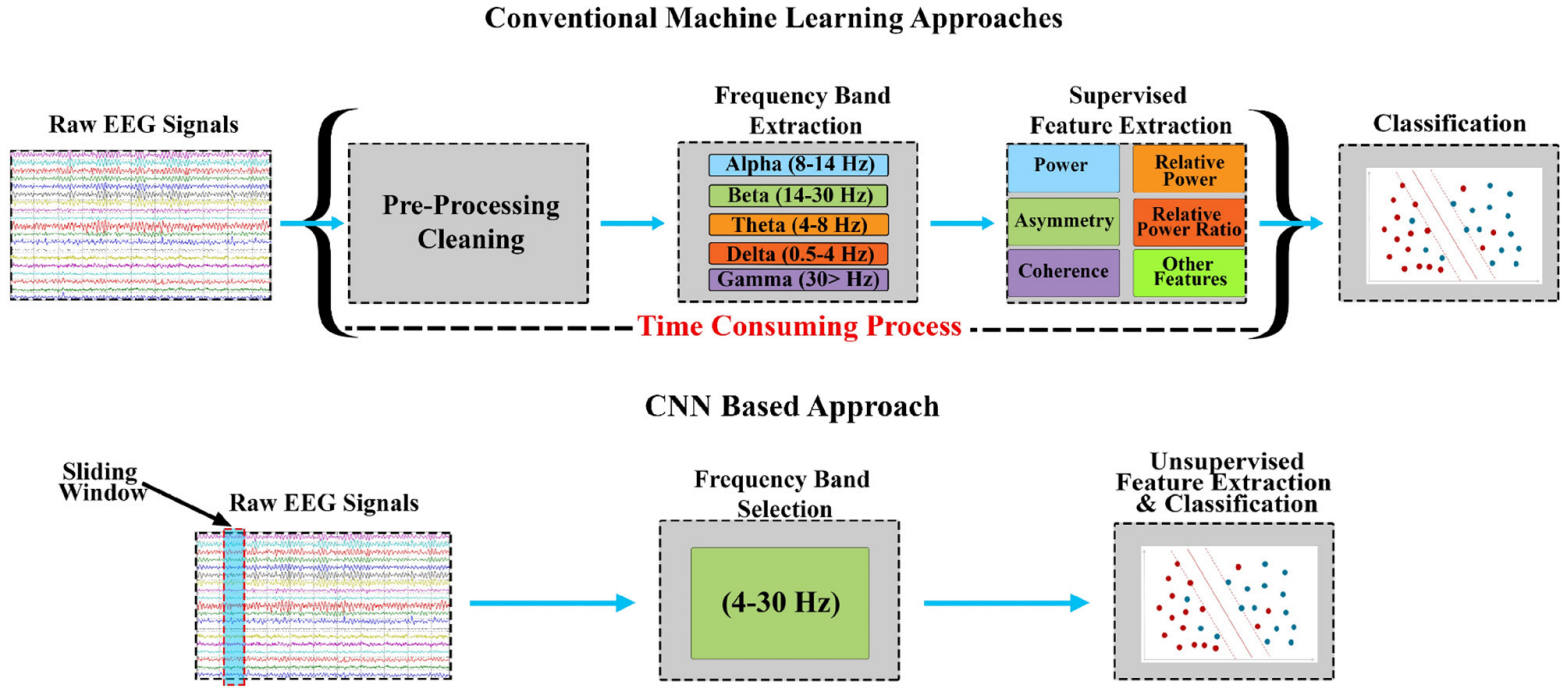
Comparison between conventional ML-based stress assessment methods and CNN-based real-time stress assessment methods. The approach eliminates the pre-processing cleaning, individual bands extraction, and supervised feature extraction phases (Naqvi et al., 2020).

Figure 1 demonstrates the conventional process flow that was followed in this research.

#### 2.1.1. Signal Filtration

Manual pre-processing is not possible for a system that operates in real-time. Therefore, cleaning of signals has to be automated and signal filtration can be used for this purpose. In the case of ML, different frequency bands are extracted i.e., delta(0.5–4), theta(4–8), alpha(8–14), beta(14–30), and gamma(>30). In contrast, in the case of DL approaches, filtration can be reduced to a single collection of bands from which features can be extracted. A Butterworth filter was used in this research which extracts the frequency bands that include theta, alpha, and beta, mainly (4–30 Hz).

#### 2.1.2. Feature Extraction for ML

The most important parameter in ML is the process of feature extraction. The quality of feature extraction affects the performance of the final trained model. Identification of significant features is a crucial and tedious task that is performed by experts. If the features do not contain discriminating properties, then it becomes difficult to perform classification among the features. Absolute power from each location with respect to five frequency bands was used in this study. There are 783 features that include power, relative power, relative power ratio, coherence, and asymmetry with 95, 95, 114, 224, and 255 features, respectively. A few additional features derived from these absolute powers are described as follows:

- Coherence:

Coherence is similar to Pearson correlation coefficient, it is calculated among all the electrodes between intra-hemisphere locations and also at homologous positions. Furthermore, it is calculated over all the frequency bands that are extracted, defined in 2.1.1. It represents the connectivity or the association between any two locations of the brain. Coherence is defined as the ratio between the cross-spectra $|H_{uv}{|}^{2}$ and the product of the auto-spectra of the two signals (|*H*_*u*_||*H*_*v*_|). Mathematically, it is represented as shown in Equation (1):


(1) $$\begin{array}{r} Coherence=\frac{|H_{uv}{|}^{2}}{\left(\left|H_{u}||H_{v}\right|\right)} \end{array}$$

- Amplitude asymmetry:

It is computed as a ratio between the difference of amplitude with the normalized sum of their amplitudes. The mathematical expression for amplitude asymmetry is demonstrated as below in Equation (2):


(2) $$\begin{array}{r} Asymmetry=\frac{M-N}{M+N} \end{array}$$

- Relative power ratio:

In order to analyze the significant and dominant frequency over one another. Different relative power ratios are computed for every electrode that includes delta/theta, delta/alpha, delta/beta, theta/alpha, theta/beta, and alpha/beta are computed. In this manner, 114 ratios for every subject is computed. Expression is demonstrated in Equation (3)


(3) $$Relative\;Power\;Ratio=\frac{PowerinBand}{\;Total\;Power}\;\times \;100$$

- CNN features: The features that are obtained in CNN are derived from the input from the proceeding layer and largely depend on the weights associated with the current layer. The features are extracted during the phase of training and such process is entirely automatic and no human intervention is required to govern it. The nature of the features changes with the change in hyperparameter that involve various factors. These factors are tested on different conditions in order to obtain the most efficient feature set for classification.

### 2.2. Benchmarking

In order to compare different models with each other based on their processing-time DT, SVM and LR are used in this study that uses hand-engineered features, whereas CNN is used as one of the DL technique. Fine trees are used as a variant of DTs whereas Gaussian kernel is used for SVM. The critical comparison is between the concept of using hand-engineered features and the process of the automatic feature extraction that is an essential and pivotal concept of DL.

## 3. Description of Dataset

**Figure 2:**
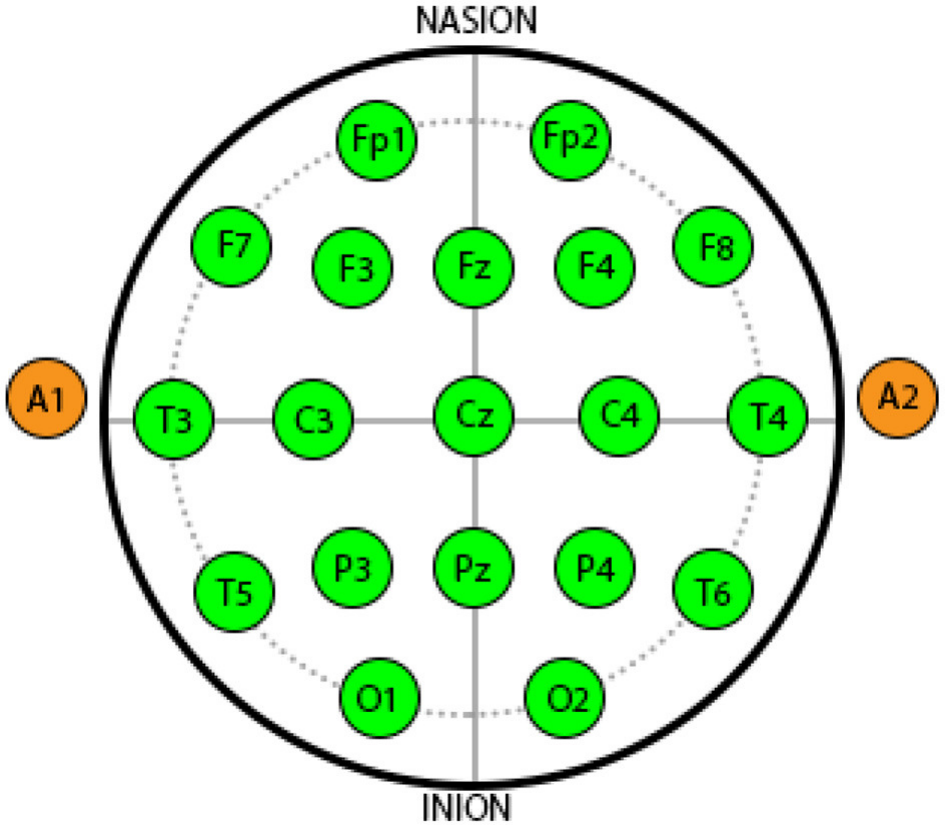
Electroencephalography (EEG) data from 19 Electrode locations according to 10–20 system international (Naqvi et al., 2020).

The dataset used in this research was collected and published by Xia et al. (2018), Subhani et al. (2016), in order to classify stress levels. The setup consist of Net Amp 300 amplifier that use 128 electrodes having Cz as reference. Less than 50 K ohm impedance was set for all the electrodes. The recording was performed over 500 samples per second with a notch filter to avoid the line noise. The stress was induced using computer-based mental arthimetic tasks, following the Montreal imaging stress task (MIST) (Dedovic et al., 2005). The protocol contains 3 sessions: relaxation, four level of stress, and control. In this research, 19 electrodes were used after selection based on 10–20 montage with an average mastoid reference (labeled as Fp1, Fp2, F3, F4, F7, F8, C3, C4, T3, T4, T5, T6, P3,P4, O1, O2, Fz, Cz, and Pz). Figure 2 shows the different locations of the electrodes. The details of data set are presented in Subhani et al. (2016) and Xia et al. (2018).

The training algorithms for the conventional ML approaches are presented in SVM (Al-shargie et al., 2015), DT (Naqvi et al., 2020), and LR. The CNN is trained using Adam optimizatrion over 50 epochs while a batch-size of 2,000 was set for maximum effectiveness with validation data. The validation of the model was performed after every 50 iterations to identify the stopping criteria and avoid the overfitting of the model. After each epoch training, the training data is specified to shuffle hence adding an extra caution regarding the overfitting. The CNN model consisted of two convolutional layers for feature extraction, pooling for extracting signification features, and leaky-relu to avoid anomaly data points or for feature cleaning. The details for training, validation, and testing are presented in Naqvi et al. (2020).

## 4. Results and Discussion

This study experimentally investigates the appropriate approach for mental stress assessment in real-time. A comparative analysis is presented between ML and DL approaches, to highlight their applicability over the objectives of this study. Different mental stress assessment pipelines are designed based on various factors to ensure high-accuracy results within the least duration of classification time.

The methodology included signal filtration, feature extraction in case of ML, and classification model. The process of signal filtration was the most expensive during the experimentation and analysis. The conventional ML approaches that were used for benchmarking required various filters to extract specific bands for the consideration shown in Figure 1. It is important to identify the major factors that can influence the mental stress assessment for better accuracy and timing. As discussed in Section 2, the mental stress assessment was affected by smaller windows and alpha asymmetry (AAS) labels. The accuracy of the trained models was also improved by the utilization of the sliding window approach. The sliding window approach enabled the investigation of mental stress signatures within EEG signals. SVM, LR and DT were used as ML models with batch-wise EEG signals and smaller windows. ML models performed well with smaller windows that have been labeled with the help of AAS during training. The models achieved an accuracy of 84% each which is demonstrated in Table 1. CNN was also used in comparison with ML techniques with smaller windows and AAS being a label. The combination provided optimal outcomes from CNN and it acheived an accuracy higher than the three ML techniques as shown in the Table 1.

**Table 1:** Performance comparison for real-time stress assessment.

**Performance/Techniques**	**CNN**	**DT**	**LR**	**SVM**
Accuracy	96%	84%	84%	84%
Sensitivity	95%	91%	78%	78%
Specificity	97%	71%	90%	90%

**Figure 3:**
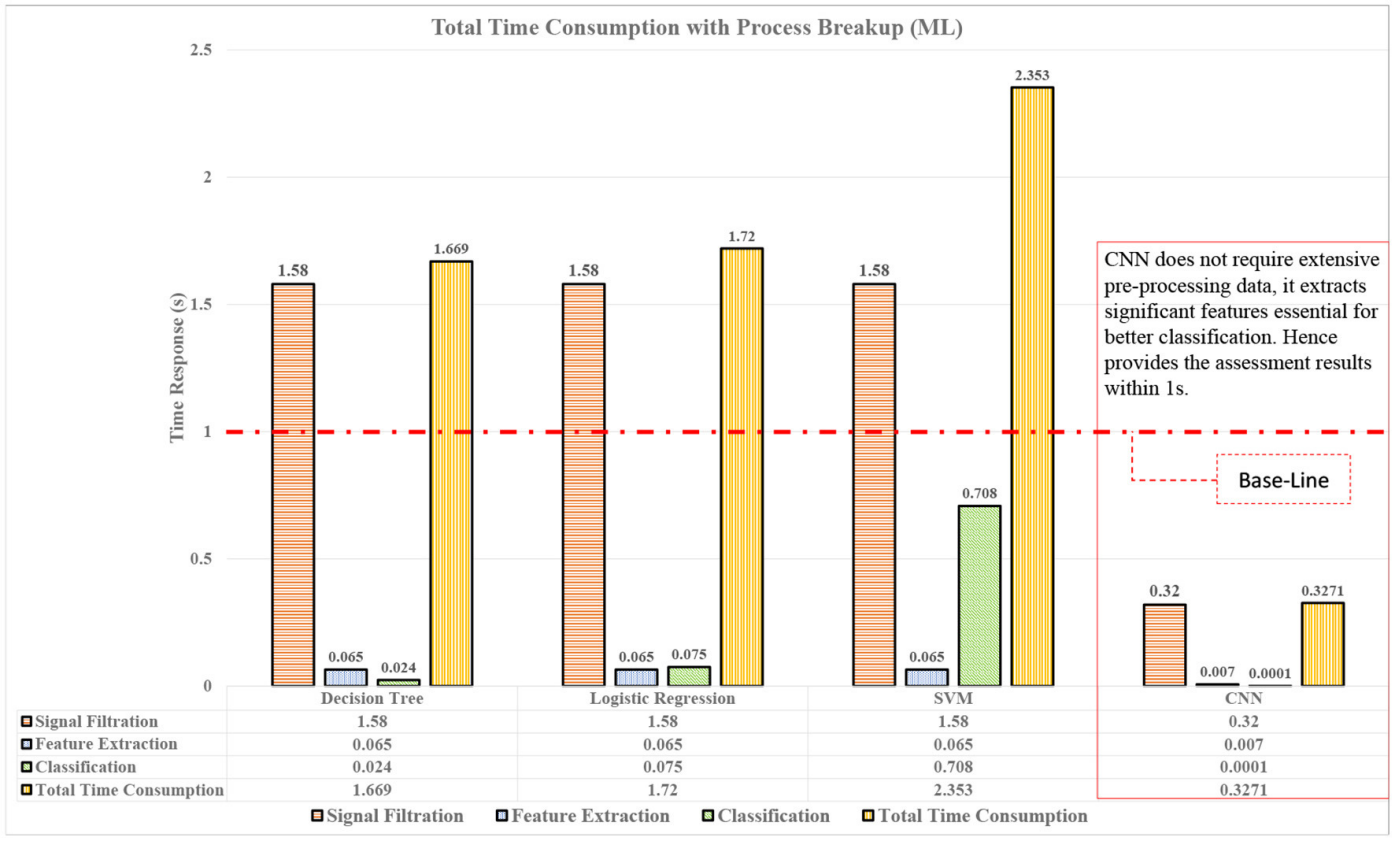
Performance comparison of techniques in terms of assessment time for real-time applications.

**Figure 4:**
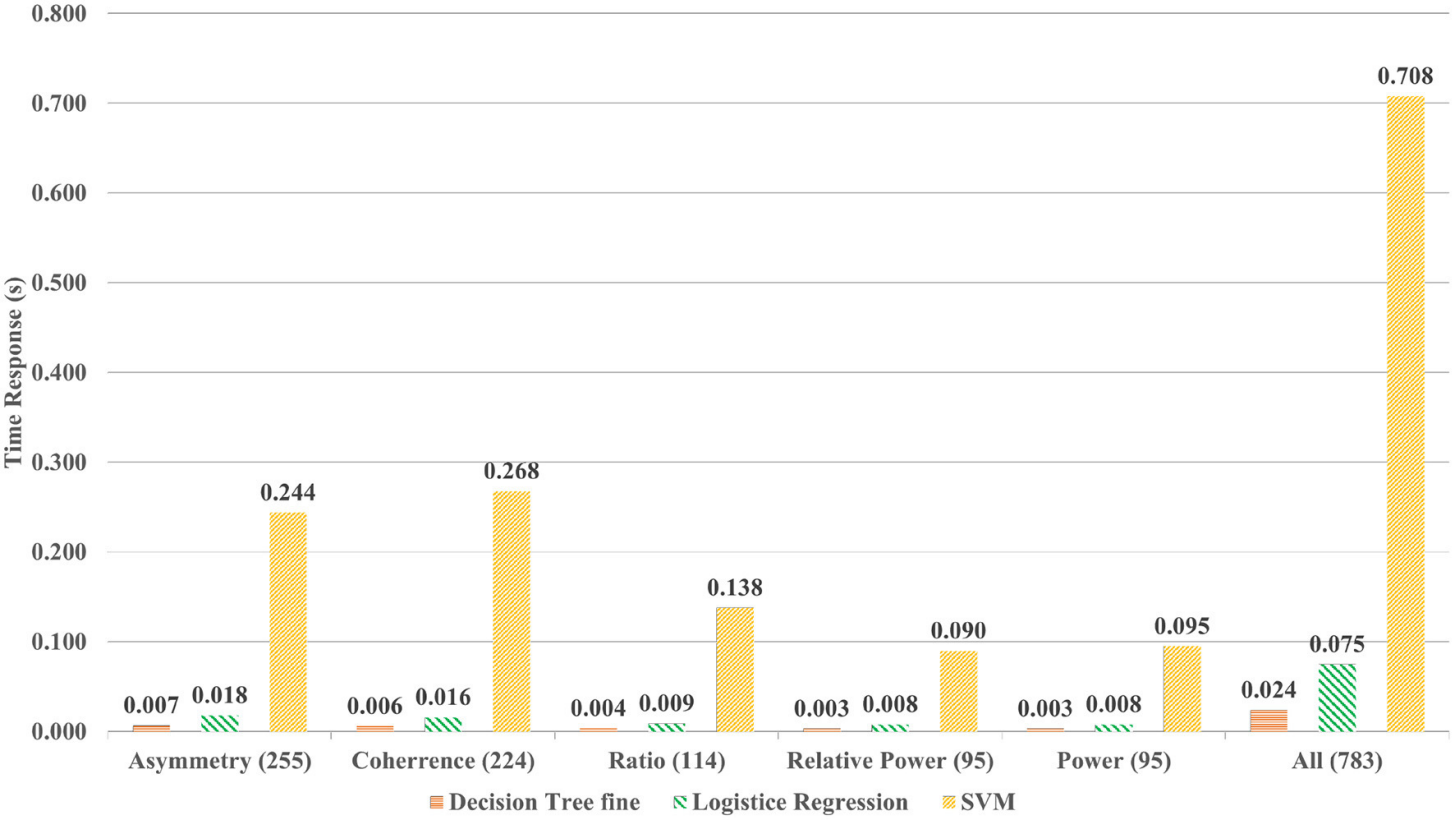
Classification time of algorithm is proportional to the quantity of the features.

The accuracy is the first milestone that is achieved by using smaller windows with AAS labels. The second objective of the study is to analyze the time duration for each intelligent model for providing the results or classification time. The classification time will justify the use of a particular model for mental stress assessment for real-time. Time was computed for each process present in the assessment pipeline to highlight the contribution for every process for classification. The assessment pipelines include three major processes namely, signal filtration, feature extraction, and finally the process of classification, which is briefly demonstrated in Figure 3. Time consumption based on different features is also computed and visualized in Figure 4, so that the effect of every feature being computed can also be highlighted and its possible effect over the classification by the model.

The quality or the characteristics of the features greatly influence the processing of the model. Different features are extracted as discussed in Section 2.1.2, from these features, various interpretation has been made that could justify the time consumption during classification. The models were trained over various settings and fine-tuned for better classification. In real-time, less time consumption processes are needed and classifiers were also compared over their consumption time. CNN performed the whole process in 0.32 s, that was less than any other technique used as shown in Figure 3. CNN was not only able to process the data much faster but also with the highest accuracy and produced results with 96% accuracy with respect to other techniques used.

Additionally, it was hypothesized that the high variation in SD and variance will affect the time consumption of the classifier. In Figure 3, it is shown that different processes consume different amounts of time. The most time-consuming process is the signal filtration that was reduced with the CNN-based method. In order to find the justification of time consumption for ML techniques, different quantities of features are tested. In Figure 4, the effect of the number of features over the model classification is visualized, which proves that the time consumption of ML techniques is greatly influenced by the number of feature. It is observed that with an increase in feature set, the properties and characteristics of the data are also affected and by this, the features can become more non-linear. Therefore, in order to resolve the non-linearity of the data, high order kernels are needed specifically for SVMs. The order of the kernels becomes a serious issue during working with ML techniques. Low order models are not able to perform well but higher order kernel models produce classification with high accuracy but will consume an enormous amount of time as compared to CNN.

**Figure 5:**
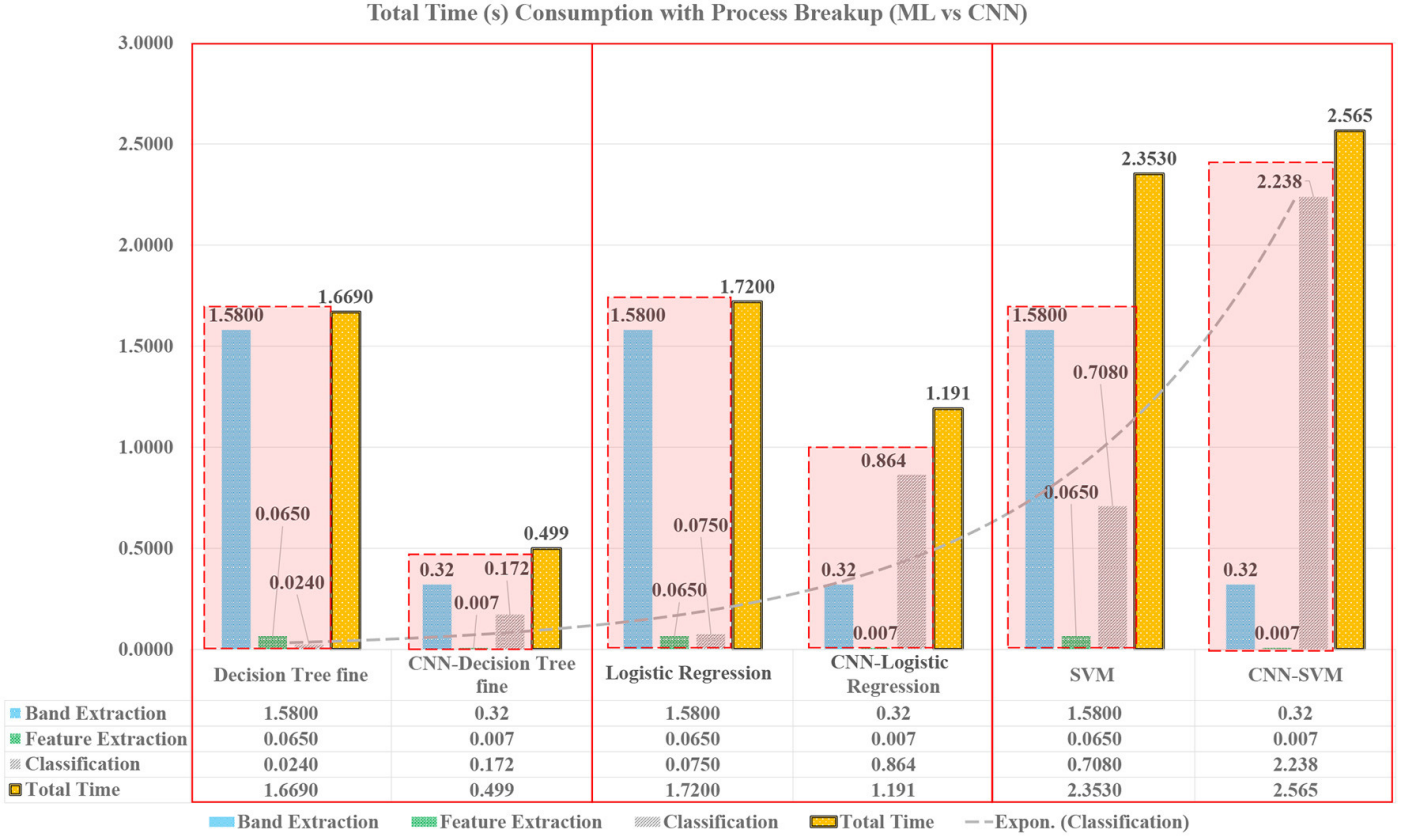
Time consumption of ML algorithms using supervised features as compared to features from CNN in terms of signal filtration, feature extraction, classification, and total time.

**Figure 6:**
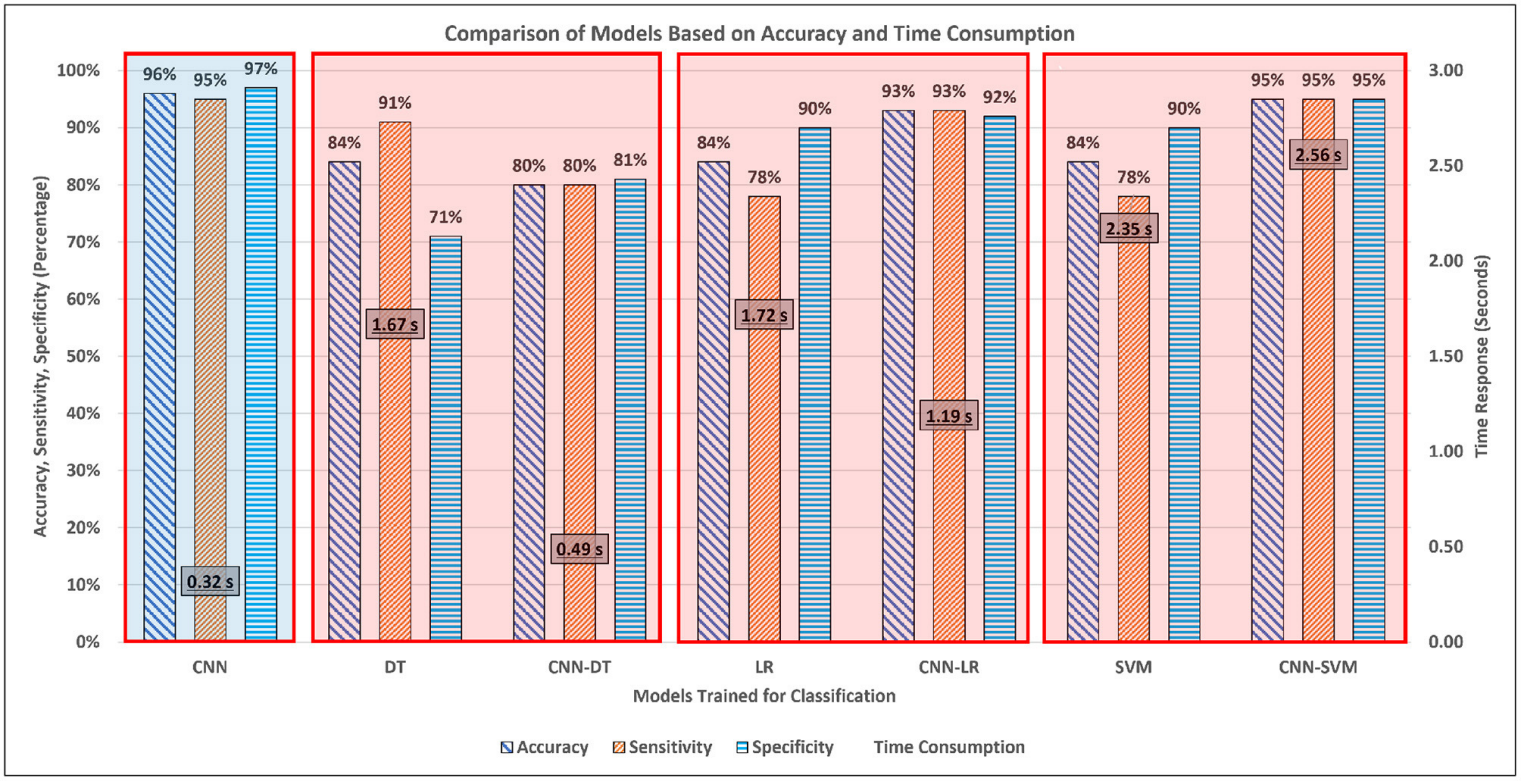
Consolidated accuracy and time consumption of ML algorithms using supervised features as compared to features from CNN in terms of signal filtration, feature extraction, classification, and total time.

In order to verify the performance benchmarking and validate the assessment accuracy vs. time consumption using CNN, a comparison is also performed using the features extracted during the training phase of CNN. The ML algorithms are trained using the CNN extracted features and tested for assessment accuracy. The performance accuracy, sensitivity, and specificity are shown in Figure 5. It was observed that the overall performance of ML algorithms improved using the CNN extracted features. However, since there were more than 3,000 features, it was also observed that the ML algorithms required more time compared to CNN as shown in Figure 5. In particular, the complexity and number of features made the SVM consume the highest time among all the algorithms. Figure 6 provides a consolidated insight with accuracy and time consumption of the models for classification of mental stress through EEG signals. CNN based assessment pipeline is found to be the most suitable technique to be used for real-time stress assessment because it provides the highest accuracy with least time consumption. Whereas, other approaches lack in providing accurate results or consume more time for assessment.

## 5. Conclusion

In this study, the behavior of ML techniques is analyzed in order to evaluate the suitability of the mental stress assessment algorithm for wearable devices. The evaluation is performed based on the computation time of the algorithm for real-time stress assessment using EEG. The assessment using conventional ML algorithms were based on pre-processing of EEG signals, band extraction, feature extraction, and classification, while the CNN based approach was applied on raw EEG signal with automatic feature extraction and assessment. It was observed the CNN, along with resulting in higher accuracy, was the most time efficient approach as compared to a decision tree, logistic regression, and support vector machines. This was due to the fact that CNN does not require a separate feature calculation step in addition to having the ability to have stress assessment on raw EEG data. The automatic features extracted during the CNN training phase were verified using the ML approaches that resulted in higher accuracy as compared to previously calculated features. This work shows that CNN is suitable for the implementation of mental stress assessment in wearable devices as it can process raw EEG data and perform stress assessment in real-time with high accuracy.

## Data Availability Statement

The data analyzed in this study is subject to the following licenses/restrictions: The data is the property of the Centre of Intelligent Signal and Imaging Research, Universiti Teknologi PETRONAS, Malaysia. Requests to access these datasets should be directed to Dr. Ahmad Rauf Subhani, raufsubhani@gmail.com.

## Ethics Statement

The studies involving human participants were reviewed and approved by the Ethics Commission in Hospital Universiti Sains Malaysia, Malaysia. This study has used the Data presented in Reference: https://doi.org/10.1016/j.bspc.2018.06.004. The patients/participants provided their written informed consent to participate in this study.

## Author Contributions

UA-S, SAlf, and SN: conceptualization. UA-S, MM, SAlf, and SN: methodology. SN and MM: software and data curation. UA-S, SAlf, and SAA: validation and resources. SAlf, SN, and MM: formal analysis. UA-S, MM, and SAlf: investigation. UA-S, SN, MM, SAlf and SAli: writing—original draft preparation. UA-S and SAlf: writing—review and editing and supervision. SN, MM, and SAlf: visualization. UA-S: project administration and funding acquisition. All authors contributed to the article and approved the submitted version.

## Funding

This research work was funded by Institutional Fund Projects by the Ministry of Education, Saudi Arabia under grant no. (IFPRC-118-135-2020).

## Conflict of Interest

The authors declare that the research was conducted in the absence of any commercial or financial relationships that could be construed as a potential conflict of interest.

## Publisher's Note

All claims expressed in this article are solely those of the authors and do not necessarily represent those of their affiliated organizations, or those of the publisher, the editors and the reviewers. Any product that may be evaluated in this article, or claim that may be made by its manufacturer, is not guaranteed or endorsed by the publisher.
